# Partial blue light blocking glasses at night advanced sleep phase and reduced daytime irritability, disruptive behavior and improved morning mood, but did not alter salivary melatonin secretion in Japanese male schoolchildren

**DOI:** 10.1371/journal.pone.0332877

**Published:** 2025-10-30

**Authors:** Naoya J. Maeda-Nishino, Ryohei Yoshimoto, Taisuke Ono, Shintaro Chiba, Seiji Nishino

**Affiliations:** 1 Sleep and Circadian Neurobiology Laboratory, Stanford University, School of Medicine, Palo Alto, California, United States of America; 2 RIKEN Center for Brain Science, Wako, Saitama, Japan; 3 Graduate School of Arts and Sciences, The University of Tokyo, Meguro-ku, Tokyo, Japan; 4 Department of Geriatric Medicine/Sleep Medicine Center, Kanazawa Medical University School of Medicine, Kahoku, Ishikawa, Japan; 5 Department of Otorhinolaryngology, Jikei University School of Medicine, Tokyo, Japan; 6 Ota Memorial Sleep Center, Ota General Hospital, Kawasaki, Kanagawa, Japan; Charite Medical University Berlin, GERMANY

## Abstract

In modern society, delayed sleep patterns among schoolchildren present challenges to academic attendance and performance. The impact of nighttime light exposure, especially blue wavelength light, on sleep delay has long been acknowledged. We investigated the effects of using partial blue light blocking glasses (JINS Screen Lens Heavy [40% cut]) on salivary melatonin levels, sleep patterns, sleep circadian phase, and daytime behavior in 39 male schoolchildren aged 10–12 who regularly wear glasses for myopia. Participants alternated between blue light blocking and standard clear lens glasses, both providing vision correction, for three hours before their habitual bedtime. The study was conducted over five weeks using a crossover design with two-week glasses-wearing sessions and a one-week washout interval between conditions. While blue light blocking glasses did not influence salivary melatonin levels, they significantly advanced the sleep phase (bedtime: 22.03 ± 0.08h vs. 22.13 ± 0.09h, p = 0.040, sleep onset: 22.26 ± 0.08h vs. 22.36 ± 0.10h, p = 0.041). The effects were more pronounced in the second week and accompanied by reduced irritability and disruptive behavior during daytime. Our results suggest that wearing blue light blocking glasses before bedtime may advance the sleep phase and improve daytime behavior in schoolchildren under real-world living conditions, warranting further mechanistic investigation.

## Introduction

People in the modern era, including young schoolchildren, are experiencing a continuous decrease in sleep duration each year [1–4]. For instance, based on a health survey conducted by the Japan Broadcasting Corporation (NHK) [5], both adults and children now sleep nearly one hour less compared to the 1960s. The same survey reports that the average bedtime for elementary school students in grades 4–6 in the 1960s was 9:20 p.m., but in 2010, it was reported to be after 10:00 p.m. [5].

This trend towards sleep loss and delayed bedtimes, especially among schoolchildren, has adverse consequences on academic attendance, performance, and social interactions [1,4].

Multiple factors, such as the demands of a busy lifestyle (schoolwork and extracurricular activities), stress and anxiety (e.g., academic pressures), and circadian/environmental influences (nighttime light exposure), likely contribute to the deterioration of sleep hygiene in schoolchildren [6]. Sleep loss and delayed circadian rhythms can lead to daytime irritability, poor concentration, and even aggressive behavior in young individuals, sometimes resulting in school absenteeism [7].

Among the factors that disrupt sleep and circadian rhythms, the increasing use of digital screens, such as smartphones, tablets, computers, and TVs, particularly during the evening, stands out [8,9]. The blue light emitted by these screens can interfere with the body’s natural sleep-wake cycle, making it more challenging to fall asleep and wake up in the morning, and disrupt daytime activities [8–10]. It is also known that children and early adolescents are more sensitive to the sleep and circadian effects of evening light exposure than older adolescents and adults [11,12].

Daytime exposure to sunlight is vital for overall human health and well-being. In terms of sleep and circadian physiology, blue light in the short-wave spectrum is particularly impactful, although sunlight encompasses the entire wavelength spectrum [13–16]. Melanopsin is a newly identified intrinsically photosensitive retinal ganglion cell (ip-RGC) and influences various non-visual responses, such as regulating the body’s circadian rhythm and mood by detecting the presence or absence of light. It relays light input to the suprachiasmatic nucleus (SCN), the master biological clock located in the hypothalamus and enhance vigilance and mood [17–19]. In mammals, melanopsin in the retina responds to blue light (~ 480 nm) [20] and inhibits melatonin secretion at night. The peak of melatonin suppression in humans is reported to be around 460 nm [21,22]. Melatonin is a hormone produced by the pineal gland in the brain and plays a crucial role in regulating sleep-wake cycles and circadian rhythm [23]. It is also known that melanopsin signals transmitted through the SCN directly activate various brain regions, enhancing both vigilance and mood [24]. Therefore, minimizing exposure to light, particularly short-wave blue light, during the night is advised to avoid disrupting sleep and circadian rhythms [23,25–28]. However, this can be challenging in our modern society with abundant artificial light exposure at night.

Numerous blue light-blocking glasses have been introduced. Previous studies indicate that light exposure diminishes melatonin secretion and disrupts sleep and circadian rhythms in both adults and children [29], and blue light-blocking glasses can reverse these effects by preventing melatonin inhibition and sleep impairments [10,30].

We therefore hypothesize that (1) wearing blue light-blocking glasses in the evening reduces nighttime stimulation of melanopsin, leading to earlier sleep onset and improved sleep, either through increased melatonin secretion or decreased direct brain stimulation (compared to wearing standard clear lenses). (2) Additionally, improved sleep is expected to positively influence and enhance daytime behavior under conditions of reduced blue light exposure in the evening.

In this study, we examined the impact of partial blue light-blocking glasses (JINS Screen Lens Heavy [40% cut]) on salivary melatonin levels, sleep patterns, sleep circadian phase, and daytime behavior (via actigraphs and questionnaires). This study involved 39 male schoolchildren aged 10–12 who regularly wear glasses for myopia and aimed to evaluate the effects of blue light-blocking glasses in various scenarios.

## Materials and methods

### Participant eligibility and recruitment

**Fig 1:**
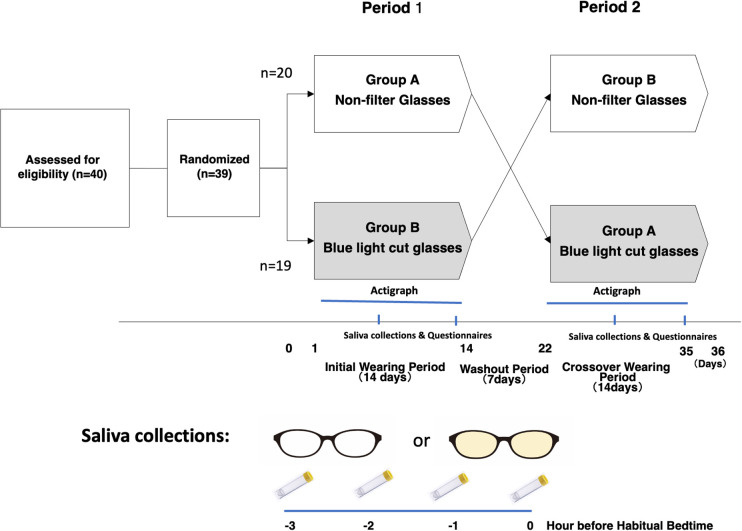
Survey design. The study design was a crossover design, with participants wearing respective glasses for 3 hours before their habitual bedtime for two weeks, separated by a one-week washout period (using their own regular glasses) between sessions. Participants were instructed to use LED display electronic devices with the blue light-cut mode (night mode, etc.) deactivated, taking into consideration its potential effect on the study results.

We recruited 40 healthy male schoolchildren aged 10–12 years old. The study was registered at UMIN-CTR (UMIN000047256) on 03/23/2022. Female schoolchildren were not included, as it is known that fist menstruation and menstruation significantly affect sleep and behavior [31]. One subject withdrew before the initiation of the data collection (Fig 1). The selection criteria included: (1i) Regular use of vision correction glasses for myopia in daily life and (2i) According to reports from parents, exposure to blue light from LED displays (such as mobile phones, tablets, and computers) for an average of one hour or more per day before bedtime. To improve compliance with wearing lenses, we enrolled only students who regularly wear glasses for myopia. In addition, we used blue light filtering glasses and regular prescription glasses, both providing vision correction, instead of overlay blue light-blocking glasses, to reduce discomfort during trails.

Participants were excluded if they: (1e) Were taking medications or supplements that affect sleep, autonomic nerves, or the central nervous system; (2e) Had irregular sleeping habits or a history of or were suffering from sleep disorders, psychiatric diseases, immune/allergic diseases, metabolic disorders, corneal disorders, retinal disorders, dry eye, or eye strain; (3e) Were taking medications or required drug treatment for hay fever; (4e) Had infectious diseases as stipulated in the Infectious Diseases Control Law and blood-borne diseases (HBV, HCV, HIV, syphilis). We assessed the degree of sleep disturbance in individual participants using the Elementary School Children’s Sleep Questionnaire by Kuwada et al [32]. This questionnaire evaluates sleep status over the last month using nine categories (I. RLS; Restless legs syndrome, II. Excessive daytime sleepiness, III. Obstructive sleep apnea syndrome, IV. Daytime behavior, V. Morning symptoms, VI. Sleep duration, VII. Insomnia/rhythm, VIII. Sleep habits, IX. Sleep rhythms on holidays), with a total of 38 questions, each with six scoring grades (1) Not at all applicable, (2) Not applicable, (3) Somewhat applicable, (4) Somewhat applicable, (5) Applicable, (6) Very applicable.

We explained the study’s aim to the participants: to investigate the influences of different types of lenses worn before bedtime on sleep and daily behaviors. However, the characteristics of each lens and the predictability of the results were not addressed. We also asked parents to monitor and record whether participants wore the correct glasses for at least three hours before their habitual bedtime. The trial period was scheduled from May through July to avoid the start of the new school year in April (in Japan), summer holidays, and major events such as sports festivals and off-campus learning activities in Fall. As the sunset during the trial period was between 6:40 and 6:50 pm and we anticipated that participants would go to bed around 10:00 pm, we set the duration for 3 hours. Since the volunteers are elementary school students, we aimed to avoid placing a burden on them. Additionally, we needed to steer clear of any major school events that could interfere with the testing. We concluded that four to five weeks would be the maximum duration for the study. Since the trial lasted a total of five weeks, there were no restrictions on eating, drinking, or posture.

This research was conducted in compliance with the research protocol and the “Ethical Guidelines for Life Sciences and Medical Research Involving Human Subjects” (Ministry of Education, Culture, Sports, Science and Technology, Ministry of Health, Labor and Welfare, and Ministry of Economy, Trade and Industry Notification No. 1, 2021). Ethical approval was obtained from the Ethical Review Committee of Ota Sleep Science (#20028), and all participants and their parents provided written informed consent. The first participant was recruited on 5/17/2022 and the trial ended on 7/7/22 (Fig 1).

### Study design and intervention

The study employed an exploratory design with a prospective, randomized, sham-controlled, single-blind crossover approach. Participants were provided with custom-made regular glasses (JINS Standard Clean Lens) and blue light-blocking glasses (JINS Screen Lens Heavy [40% cut]) with correcting vision based on the degree of myopia of each participant, both fitted into frames matching their usual glasses (Fig 1). The transmittance spectrum of blue light cut and normal lenses are shown in S1 Fig.

The study assessed the effects of wearing partial blue light-blocking glasses for a duration of 3 hours before their habitual bedtime. Participants alternated between the two types of glasses over two-week sessions, with a one-week washout period (using their own regular glasses) between sessions. Random assignment of subjects ensured an equal ratio of participants in the “Non-filter glasses pre-wear group: Group A” and the “ Blue light-blocking glasses pre-wear group: Group B” at a 20:19 ratio.

Participants were instructed to use LED display electronic devices with the blue light- blocking mode (night mode, etc.) deactivated during the study, to avoid any potential influence on the study results.

Saliva samples were collected using Saliva Collection Straws (Saliva Collection Aid) and transferred to saliva collection tubes (Cryovial, 2 ml, SalivaBio) on the day before the start of wearing the spectacles, and on the 7th, 14th, 28th, and 35th days of the study. Participants collected 0.5 ml of saliva in Cryovial containers using the saliva collection straws at −3, −2, −1, and 0 hours before their habitual bedtime (Fig 1). The collected specimens were stored in freezers in the participants’ homes and later transported to a laboratory, where they were stored at −80°C until the melatonin concentration in saliva was measured using the Melatonin test kit (SALIMETRICS ASSAY, Salimetrix, LLC. Carlsbad, CA).

To monitor sleep patterns, a lightweight waist-worn actigraph MTN-221 (Acos, Co., Ltd. Nagano, Japan) was used. Subjects wore the actigraph on their abdomen with a designated belt approximately 3 hours before bedtime, and they continued their usual activities until bedtime (but removed the actigraph while taking a bath). The actigraph was worn throughout the bedtime and removed upon waking the next day. The subjects also maintained a daily sleep diary to record bedtime and wake-up time in conjunction with the actigraph recordings.

Sleep and wakefulness were analyzed using SleepSign®-Act Ver. 2.0 (Kissei Comtec Co., Ltd, Matsumoto, Japan), which employs an algorithm based on activity and posture data collected by a small, lightweight actigraphy device and performs a series of linked calculations [33–35]. This actigraph, positioned on the trunk, detects various postures (Standing, Inverted, Spine, Prone, Left lateral decubitus, Right lateral decubitus) (S2 Fig) and provides reliable evaluations of Into-Bed Time, Out-of-Bed Time, which are characteristics that most wrist-worn actigraphs struggle to measure accurately. In addition to Into-Bed Time and Out-of-Bed Time, the algorithm automatically calculates Sleep Onset Time, Total Sleep Time, Sleep Latency, Wake after Sleep Onset, Sleep Efficiency, Final Wake up Time, Out-of-Bed Time, and Bed out Latency. One of the authors further verified (blinded to session information) the Bedtime and Out-of-Bed Time for each recording day with the assistance of the individual’s daily sleep log (i.e., Sleep Onset and Wake up Time) recorded each day. Data from weekdays (Monday to Friday) for each week were analyzed and used for comparisons.

In order to evaluate changes in sleep and daytime behavior during trial periods, 23 questions (each with six options) selected from (JSQ-ES) [32], were administered on the 8th, 15th, 29th, and 36th days after the end of wearing the spectacles (Fig 1, S1 and S2 File). We asked the participants to record their condition over the past few days. These questions cover various aspects of sleep and behavior, including excessive daytime sleepiness, daytime behavior, morning symptoms, insomnia/rhythm, holiday sleep rhythm, and chronotype on the day prior to completing the questionnaire.

## Statistical analysis

Statistical analysis was conducted using JMP16 (Cary, NC, USA) for various parameters, including salivary melatonin levels, actigraph data, and scores related to sleep and daytime behavior. Homogeneity of variance was assessed using Levene’s test. When normality and homogeneity of variance were not recognized in the data, nonparametric analysis was performed. Melatonin levels were analyzed using a two-way repeated measures ANOVA, while actigraph data and sleep/daytime behavior scores were analyzed using the nonparametric Wilcoxson signed-rank test, as normality was not confirmed in most of the dataset. The threshold for statistical significance was set at p < 0.05 (two-sided). All data are presented as mean ± SEM. A priori power analysis estimates the requirement of 27 cases for the Wilcoxon signed-rank test, using an effect size (0.5), a power value of 0.8, and a two-tailed significance level of p < 0.05.

## Results

### Sleep and lifestyle of participants

The mean scores (±SD and range) of the JSQ-ES for the 39 participants were as follows: I. Restless legs syndrome (7.1 ± 1.8, 6–13), II. Excessive daytime sleepiness (8.7 ± 4.0, 5–19), III. Obstructive sleep apnea syndrome (7.8 ± 3.8, 3–16), IV. Daytime behavior (6.6 ± 2.1, 5–11), V. Morning symptoms (3.8 ± 1.4, 3–8), VI. Sleep duration (6.4 ± .0, 4–11), VII. Insomnia/rhythm (9.9 ± 4.6, 4–20), VIII. Sleep habits (4.1 ± 3.1, 2–12), IX. Sleep rhythms on holidays (8.9 ± 3.3, 4–15). Based on these results, we confirmed that none of the participants exhibited significant sleep disorders.

Additional observations included: 12.8% of participants reported tooth grinding, 5.1% experienced nocturnal behaviors, 2.6% fell asleep while watching TV, 5.1% went out after 8 p.m., and 100% reported eating breakfast daily. Furthermore, 64.1% spent more than one hour using LED-emitting devices just before bedtime, and 10.3% occasionally consumed caffeine after 7 p.m.

### Salivary melatonin levels

**Fig 2:**
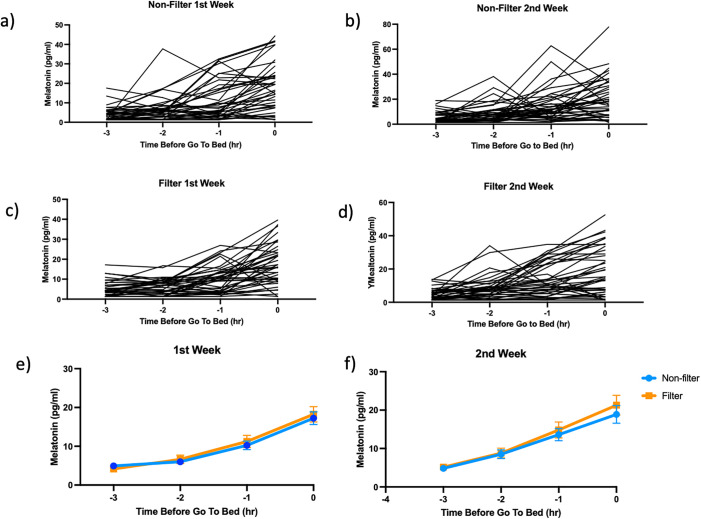
Salivary melatonin data of all individuals in weeks 1 and 2 of both Non-Filter and Filter sessions (a-d), and a comparison of average salivary melatonin levels between Non-Filter and Filter sessions in weeks 1 and 2 (d, e). Saliva measurements were conducted during the 1st and 2nd weeks of each session, with saliva collected at −3, −2, −1, and 0 hours before the habitual bedtime **(a-d)**. Time-dependent increases in salivary melatonin levels were observed in most subjects in both sessions during weeks 1 and 2 **(d, e)**. The time-dependent increases in melatonin levels were significant (p < 0.0001), but no significant differences were observed between the Sessions or in the Session x Time interaction (p > 0.05, Two-way Repeated Measures ANOVA).

The salivary melatonin data for all individuals in weeks 1 and 2, both in the Non-Filter and Filter glasses wearing sessions, are presented in Fig 2 (a-d). Additionally, Fig 2 (e, f) displays the comparison of average salivary melatonin levels between the Non-Filter and Filter wearing sessions in weeks 1 and 2.

A time-dependent increase in melatonin levels, approximately four times higher than the lowest time point, was observed in most subjects from both sessions during week 1 and week 2 (Fig 2. a-d). Analysis of average salivary melatonin levels for the Non-Filter and Filter sessions revealed a significant time-dependent increase in salivary melatonin levels in both sessions during both weeks (Two-way repeated measures ANOVA, 1st week, Time effect: F = 31.6, df = 3, 74, p < 0.0001) and 2^nd^ week (Time effect: F = 29.1, df = 3, 74, p < 0.0001) (Fig 2. e, f). However, no significant differences (1st week: Time x Session: F = 0.46, df = 3, 74, p = 0.71, 2nd week: Time x Session: F = 0.12, df = 3, 74, p = 0.95) were observed between the Sessions, and there was no significant Time x Session interaction (Fig 2. e, f). These results suggest that light exposure during regular evening activities did not significantly reduce melatonin secretion, and that wearing 40% partial blue light-blocking glasses for 3 hours before bedtime did not restore salivary melatonin secretion.

These results indicate that that wearing 40% partial blue light-blocking glasses for 3 hours before bedtime did not alter melatonin secretion observed with Non-filter session.

### Actigraph sleep measures

Since no statistical differences were observed in any sleep parameters between Group A and B during both Non-Filter and Filter sessions (see Fig 1 for group identification), the data for Group A and B in each Non-Filter and Filter session were combined and analyzed accordingly. We noticed that Japanese 10–12 year old school children in our study had very short sleep duration and low sleep efficacy, such as 358.53 minutes 71.81% in Non-Filter and 356.03 minutes and 70.78% in Filter sessions (Table 1). According to self/parental reports, sleep duration is much longer and estimated to be around 8.4 hours in Japanese children aged 10–12 years old [5], values roughly correspond to the Total Time in Bed in our study (502.37 minutes in Non-Filter sessions and 504.69 minutes in Filter sessions), suggesting that the self-reported sleep time may reflect the Total Time in Bed. It is also possible that the automatic sleep scoring used [33–35] was too sensitive in detecting awakenings in school children, resulting in shorter total sleep time and lower sleep efficiency. However, our actigraph has the advantage of accurately detecting sleep phases through posture data, and sleep phase advancement is one of our hypotheses.

**Table 1:** Analysis of 2-week average of sleep actigraph data between Non-filter and Filter sessions.

		Total Sleep Time(TST)	Sleep Latency(SL)	Wake After Sleep Onset(WASO)	Number of Awakenings(NOA)	Sleep Efficiency(SE)
**Mean±SEM**	**Non-Filter**	358.53 ± 7.67	14.11 ± 1.98	120.30 ± 7.34	15.16 ± 0.47	71.81 ± 1.43
	**Filter**	356.03 ± 6.70	13.65 ± 1.70	127.67 ± 7.70	15.76 ± 0.56	70.78 ± 1.51
**p-value**	**wilcoxon**	0.68	0.48	0.13	0.26	0.26
		**Average Awakening Duration**	**Awakenings Longer Than 8 Minutes**	**Number of Position Changes**	**Into-Bed Time**	**Sleep-Onset Time**
**Mean±SEM**	**Non-Filter**	7.78 ± 0.30	6.85 ± 0.46	25.69 ± 1.13	22.13 ± 0.09	22.36 ± 0.10
	**Filter**	7.93 ± 0.30	7.15 ± 0.48	26.63 ± 1.34	22.03 ± 0.08	22.26 ± 0.08
**p-value**	**wilcoxon**	0.38	0.28	0.25	0.040*	0.041*
		**Final Wake Time**	**Out-of-Bed Time**	**Sleep Period Time(SPT)**	**Total Time in Bed(TIB)**	**Bed Out Latency(BOL)**
**Mean±SEM**	**Non-Filter**	6.35 ± 0.11	6.47 ± 0.11	478.83 ± 5.09	500.40 ± 5.69	7.46 ± 0.54
	**Filter**	6.32 ± 0.09	6.45 ± 0.10	483.69 ± 3.87	505.05 ± 5.17	7.70 ± 0.69
**p-value**	**wilcoxon**	0.74	0.84	0.11	0.09	0.85

A significant advancement in Into-Bed Time and Sleep-Onset Time was observed between Non-Filter and Filter sessions in 2-week average.

Analysis of the average two-week sleep actigraph data between the Non-Filter and Filter sessions revealed a significant advancement in Into-Bed Time (22.13 ± 0.09h, vs. 22.03 ± 0.08h n = 39, W = 129.5, p = 0.040) and Sleep-Onset Time (22.36 ± 0.10h, vs. 22.26 ± 0.08h, n = 39, W = 129.0, p = 0.041) in the Filter session compared to the Non-Filter session (Table 1). The mean durations in advance are 6.0 minutes for Into-Bed Time and Sleep-Onset Time. Further analysis of the individual week data within the Filter sessions (week 1 vs. week 2) indicated that the effects began to manifest in the second week, with significant effects observed for Into-Bed Time and Sleep-Onset Time during the second week (Tables 2, 3).

**Table 2:** Analysis of Week 1 sleep actigraph data between Non-filter and Filter sessions.

		Total Sleep Time(TST)	Sleep Latency(SL)	Wake After Sleep Onset(WASO)	Number of Awakenings(NOA)	Sleep Efficiency(SE)
**Mean±SEM**	**Non-Filter**	354.00 ± 7.95	14.17 ± 1.74	122.64 ± 7.96	15.23 ± 0.55	71.25 ± 1.57
	**Filter**	356.55 ± 7.52	14.19 ± 1.64	126.79 ± 8.29	15.43 ± 0.62	70.91 ± 1.66
**p-value**	**wilcoxon**	0.59	0.65	0.27	0.56	0.64
		**Average Awakening Duration**	**Awakenings Longer Than 8 Minutes**	**Number of Position Changes**	**Into-Bed Time**	**Sleep-Onset Time**
**Mean±SEM**	**Non-Filter**	7.85 ± 0.34	6.95 ± 0.50	25.51 ± 1.31	22.17 ± 0.1	22.40 ± 0.1
	**Filter**	7.94 ± 0.33	7.10 ± 0.50	26.93 ± 1.46	22.10 ± 0.1	22.34 ± 0.1
**p-value**	**wilcoxon**	0.74	0.49	0.10	0.1	0.3
		**Final Wake Time**	**Out-of-Bed Time**	**Sleep Period Time(SPT)**	**Total Time in Bed(TIB)**	**Bed Out Latency(BOL)**
**Mean±SEM**	**Non-Filter**	6.35 ± 0.12	6.47 ± 0.12	476.65 ± 5.70	498.43 ± 6.10	7.61 ± 0.68
	**Filter**	6.39 ± 0.11	6.52 ± 0.11	483.34 ± 4.80	505.41 ± 5.98	7.88 ± 0.79
**p-value**	**wilcoxon**	0.50	0.37	0.26	0.22	0.67

No significant differences were observed between the Non-Filter and Filter sessions in Week 1.

**Table 3:** Analysis of Week 2 sleep actigraph data between Non-filter and Filter sessions.

		Total Sleep Time(TST)	Sleep Latency(SL)	Wake After Sleep Onset(WASO)	Number of Awakenings(NOA)	Sleep Efficiency(SE)
**Mean±SEM**	**Non-Filter**	363.06 ± 8.31	14.05 ± 2.55	117.95 ± 7.53	15.09 ± 0.54	72.36 ± 1.46
	**Filter**	355.50 ± 7.31	13.12 ± 2.09	128.55 ± 7.63	16.10 ± 0.60	70.65 ± 1.48
**p-value**	**wilcoxon**	0.29	0.69	0.11	0.16	0.24
		**Average Awakening Duration**	**Awakenings Longer Than 8 Minutes**	**Number of Position Changes**	**Into-Bed Time**	**Sleep-Onset Time**
**Mean±SEM**	**Non-Filter**	7.71 ± 0.31	6.76 ± 0.49	25.87 ± 1.10	22.10 ± 0.10	22.34 ± 0.11
	**Filter**	7.93 ± 0.32	7.21 ± 0.49	26.32 ± 1.31	21.98 ± 0.09	22.19 ± 0.09
**p-value**	**wilcoxon**	0.34	0.31	0.48	0.039*	0.043*
		**Final Wake Time**	**Out-of-Bed Time**	**Sleep Period Time(SPT)**	**Total Time in Bed(TIB)**	**Bed Out Latency(BOL)**
**Mean±SEM**	**Non-Filter**	6.35 ± 0.10	6.47 ± 0.10	481.01 ± 5.45	502.37 ± 5.97	7.30 ± 0.58
	**Filter**	6.26 ± 0.09	6.39 ± 0.09	484.05 ± 4.58	504.69 ± 5.55	7.53 ± 0.80
**p-value**	**wilcoxon**	0.23	0.29	0.25	0.35	0.92

A significant advancement in Into-Bed Time and Sleep-Onset Time was observed between Non-Filter and Filter sessions in Week 2.

**Fig 3:**
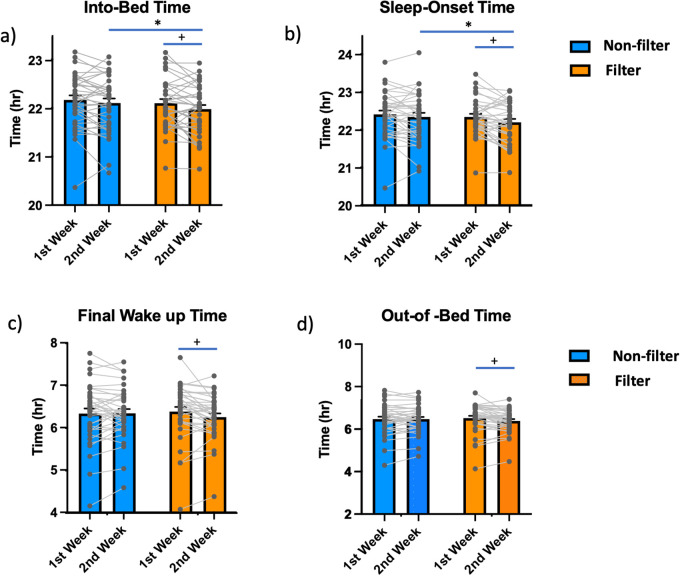
Comparisons between the 1st and 2nd weeks and between the Non-Filter and Filter sessions for Into-Bed Time, Sleep-Onset Time, Final Wake-Up Time, and Out-of-Bed Time. A significant advancement in the sleep phase was also observed between the 1st and 2nd weeks of the Filter session for Into-Bed Time **(a)**, Sleep-Onset Time **(b)**, Final Wake-Up Time **(c)**, and Out-of-Bed Time **(d)**, while no significant changes were observed between the 1st and 2nd weeks of the Non-Filter session. (* paired comparison between Non-Filter and Filter sessions, ^**+**^ paired comparison between the 1st and 2nd weeks.

Significant advancements in sleep phase ([a] Into-Bed Time, [b] Sleep-Onset Time, [c] Final Wake-up Time, and [d] Out-of-Bed Time) were observed from the 1st week to the 2nd week in the Filter session, whereas no significant changes were observed between the 1st and 2nd weeks in the Non-Filter session (Fig 3), suggesting that the use of blue light-blocking glasses contributes to the advancement of the sleep phase.

### Sleep and behavioral questionnaires

**Fig 4:**
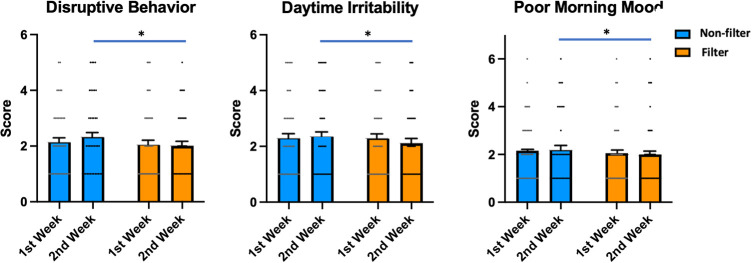
Comparisons between 1st and 2nd weeks and between the Non-Filter and Filter sessions for Daytime Irritability, Disruptive Behavior towards Siblings and Friends, and Poor Mood upon Waking. Alongside the advancement in sleep phase (Fig 3), improvements in Daytime Irritability, Disruptive Behavior towards Siblings and Friends, and Poor Mood upon Waking appeared in the 2nd week, and become significant. (^**+**^ paired comparison between the 1st and 2nd weeks.

The analysis of the average sleep and behavioral questionnaire data over a two-week period (Table 4) for the Non-Filter and Filter sessions revealed a significant decrease in Disruptive Behavior towards Siblings and Friends (2.3 ± 0.2 vs. 2.1 ± 0.2, n = 39, W = 124, p = 0.041) and Daytime Irritability (2.4 ± 0.2 vs. 2.2 ± 0.2, n = 39, W = 150.5, p = 0.015) in the Filter session. Additionally, when analyzing the individual week data of the Filter sessions (week 1 vs. week 2), it became evident that behavioral effects, in conjunction with the sleep phase advancement, started to manifest in the 2nd week (Tables 2, 3, 5, 6). These effects became significant for Disruptive Behavior towards Siblings and Friends (2.4 ± 0.2 vs. 2.1 ± 0.2, n = 39, W = 142.5, p = 0.013), Daytime Irritability (2.5 ± 0.2 vs. 2.1 ± 0.2, n = 39, W = 142.5, p = 0.017), and Poor Mood upon Waking (2.3 ± 0.2 vs. 2.0 ± 0.2, n = 39, W = 116.5, p = 0.048) during the 2nd week (Table 6). Furthermore, comparisons between the 1st and 2nd weeks showed that these parameters tended to improve in the Filter session during the 2nd week, while they worsened slightly in the 2nd week of the Non-Filter session (Fig 4).

**Table 4:** Analysis of 2-week average of sleep questionnaire data between Non-filter and Filter sessions.

		1-1 SLEEPINESS	1-2 FATIGUE	1- 3DOZING AT HOME	1 - 4DOZING AT SCHOOL	1 -5BEDTIME AFTER 11PM	1 - 6WASO >2
Non-filter	Mean±SEM	1.7 ± 0.1	1.9 ± 0.2	1.3 ± 0.1	1.1 ± 0.0	1.5 ± 0.1	1.5 ± 0.1
Filter		1.8 ± 0.1	2.0 ± 0.2	1.3 ± 0.1	1.1 ± 0.0	1.5 ± 0.1	1.6 ± 0.2
Wilcoxon	p-value	0.75	0.73	0.58	1.00	0.69	0.37
		1 - 7**IRREGULAR SLEEP**	2 − 1**DAYTIME RESTLESSNESS**	2 − 2**LESS CONCENTRATION**	2 - 3**DISRUPTIVE BEHAVIOR**	2 - 4**DAYTIME IRRITABILITY**	3 − 1**TROUBLE AWAKENING**
Non-filter	Mean±SEM	2.4 ± 0.1	2.2 ± 0.2	2.5 ± 0.2	2.3 ± 0.2	2.4 ± 0.2	2.7 ± 0.2
Filter		2.3 ± 0.1	2.2 ± 0.2	2.5 ± 0.2	2.1 ± 0.2	2.2 ± 0.2	2.6 ± 0.2
Wilcoxon	p-value	0.36	0.44	0.95	0.041*	0.015*	0.78
		3 − 2**TROUBLE GETTING OUT OF BED**	3 − 3**POOR MOOD UPON AWAKENING**	4 − 1**UNABLE TO ATTEND SCHOOL**	4 − 2**LATE FOR SCHOOL**	4 − 3**DAY AND NIGHT REVERSAL**	5 − 1** > 1 HR LATER SLEEP TIME ON WEEKENDS**
Non-filter	Mean±SEM	2.6 ± 0.2	2.2 ± 0.2	1.2 ± 0.1	1.2 ± 0.1	1.0 ± 0.0	3.4 ± 0.2
Filter		2.5 ± 0.2	2.1 ± 0.2	1.1 ± 0.1	1.1 ± 0.0	1.1 ± 0.0	3.5 ± 0.3
Wilcoxon	p-value	0.81	0.17	0.31	0.18	0.55	0.81
		5 − 2** > 1 HR LATER AROUSAL TIME ON WEEKENDS**	C-1**DIFFICULTY WAKING IN MORNING**	**C-2ALERTNESS UPON WAKING**	C-3**TIME OF INITIAL DROWSINESS**	C-4**TIME UNTIL FULL ALERTNESS**	
Non-filter	Mean±SEM	2.9 ± 0.2	1.9 ± 0.2	2.0 ± 0.2	4.1 ± 0.1	2.6 ± 0.2	
Filter		2.9 ± 0.2	1.8 ± 0.2	2.0 ± 0.2	4.3 ± 0.1	2.7 ± 0.2	
Wilcoxon	p-value	0.54	0.45	0.58	0.22	0.69	

Significant improvements in Violent Behavior and Daytime irritability were observed in the Filter sessions.

**Table 5:** Analysis of Week 1 sleep questionnaire data between Non-filter and Filter sessions.

		1-1SLEEPINESS	1-2 FATIGUE	1- 3DOZING AT HOME	1 - 4DOZING AT SCHOOL	1 - 5BEDTIME AFTER 11PM	1 - 6WASO >2
Non-filter	Mean±SEM	1.6 ± 0.1	1.9 ± 0.2	1.3 ± 0.1	1.2 ± 0.1	1.5 ± 0.1	1.6 ± 0.1
Filter		1.8 ± 0.2	2.0 ± 0.2	1.3 ± 0.1	1.2 ± 0.1	1.5 ± 0.1	1.6 ± 0.2
Wilcoxon	p-value	0.28	0.89	0.66	1.00	0.61	0.91
		1 - 7**IRREGULAR SLEEP**	2 − 1**DAYTIME RESTLESSNESS**	2 − 2**LESS CONCENTRATION**	2 - 3**DISRUPTIVE BEHAVIOR**	2 - 4**DAYTIME IRRITABILITY**	3 − 1**TROUBLE AWAKENING**
**Non-filter**	Mean±SEM	2.5 ± 0.2	2.2 ± 0.2	2.4 ± 0.2	2.3 ± 0.2	2.3 ± 0.2	2.7 ± 0.2
**Filter**		2.3 ± 0.2	2.2 ± 0.2	2.6 ± 0.2	2.2 ± 0.2	2.3 ± 0.2	2.6 ± 0.2
Wilcoxon	p-value	0.56	0.89	0.51	0.77	0.42	0.59
		3 − 2**TROUBLE GETTING OUT OF BED**	3 − 3**POOR MOOD UPON AWAKENING**	4 − 1**UNABLE TO ATTEND SCHOOL**	4 − 2**LATE FOR SCHOOL**	4 − 3**DAY AND NIGHT REVERSAL**	5 − 1** > 1 HR LATER SLEEP TIME ON WEEKENDS**
**Non-filter**	Mean±SEM	2.5 ± 0.2	2.2 ± 0.2	1.2 ± 0.1	1.2 ± 0.1	1.0 ± 0.0	3.4 ± 0.2
**Filter**		2.5 ± 0.2	2.1 ± 0.2	1.1 ± 0.1	1.1 ± 0.0	1.1 ± 0.0	3.6 ± 0.3
Wilcoxon	p-value	1.00	0.77	0.08	0.16	0.57	0.47
		5 − 2 **> 1 HR LATER AROUSAL TIME ON WEEKENDS**	C-1 **DIFFICULTY WAKING IN MORNING**	**C-2** **ALERTNESS UPON WAKING**	C-3 **TIME OF INITIAL DROWSINESS**	C-4 **TIME UNTIL FULL ALERTNESS**	
**Non-filter**	Mean±SEM	3.0 ± 0.2	1.9 ± 0.2	2.1 ± 0.2	4.2 ± 0.1	2.5 ± 0.2	
**Filter**		3.0 ± 0.2	1.8 ± 0.2	2.0 ± 0.1	4.3 ± 0.1	2.7 ± 0.2	
Wilcoxon	p-value	0.99	0.23	0.91	0.35	0.57	

No significant differences were observed between the Non-Filter and Filter sessions in Week 1.

**Table 6:** Analysis of Week 2 sleep questionnaire data between Non-filter and Filter sessions.

		1-1 SLEEPINESS	1-2 FATIGUE	1-3 DOZING AT HOME	1-4 DOZING AT SCHOOL	JSQ1 - 5C1_5BEDTIME AFTER 11PM	1-6 WASO >2
Non-Filter	Mean±SEM	1.8 ± 0.2	1.9 ± 0.2	1.2 ± 0.1	1.1 ± 0.0	1.6 ± 0.1	1.4 ± 0.1
Filter		1.7 ± 0.1	1.9 ± 0.1	1.3 ± 0.1	1.1 ± 0.0	1.5 ± 0.2	1.5 ± 0.1
wilcoxon	p-value	0.71	0.78	0.15	1.00	0.27	0.15
		1-7 **IRREGULAR SLEEP**	2-1 **DAYTIME RESTLESSNESS**	2-2 **LESS CONCENTRATION**	2-3 **DISRUPTIVE BEHAVIOR**	2-4 **DAYTIME IRRITABILITY**	3−1 **TROUBLE AWAKENING**
Non-Filter	Mean±SEM	2.4 ± 0.2	2.2 ± 0.2	2.5 ± 0.2	2.4 ± 0.2	2.5 ± 0.2	2.6 ± 0.3
Filter		2.3 ± 0.2	2.2 ± 0.2	2.4 ± 0.2	2.1 ± 0.2	2.1 ± 0.2	2.6 ± 0.2
wilcoxon	p-value	0.72	0.42	0.27	0.013*	0.017*	0.79
		3−2 **TROUBLE GETTING OUT OF BED**	3−3 **POOR MOOD UPON AWAKENING**	4−1 **UNABLE TO ATTEND SCHOOL**	4−2 **LATE FOR SCHOOL**	4−3 **DAY AND NIGHT REVERSAL**	5−1** > 1 HR LATER SLEEP TIME ON WEEKENDS**
Non-Filter	Mean±SEM	2.6 ± 0.3	2.3 ± 0.2	1.2 ± 0.1	1.2 ± 0.1	1.1 ± 0.0	2.9 ± 0.3
Filter		2.5 ± 0.2	2.0 ± 0.2	1.1 ± 0.1	1.1 ± 0.0	1.1 ± 0.0	2.9 ± 0.2
wilcoxon	p-value	0.36	0.048*	0.56	0.18	0.16	0.74
		**5−2 > 1 HR LATER AROUSAL TIME ON WEEKENDS**	C-1**DIFFICULTY WAKING IN MORNING**	**C-2ALERTNESS UPON WAKING**	C-3**TIME OF INITIAL DROWSINESS**	C-4**TIME UNTIL FULL ALERTNESS**	
Non-Filter	Mean±SEM	2.9 ± 0.3	1.9 ± 0.2	2.0 ± 0.2	4.1 ± 0.2	2.6 ± 0.2	
Filter		2.9 ± 0.2	1.8 ± 0.2	1.9 ± 0.2	4.2 ± 0.2	2.7 ± 1.2	
wilcoxon	p-value	0.88	0.66	0.51	0.16	0.95	

Significant improvements in Violent Behavior and Daytime Irritability, Poor Mood Upon Awakening were observed in the Filter sessions.

## Discussion

Our results indicate that wearing partial blue light-blocking glasses advances sleep onset and enhances daytime behavior in schoolchildren. These effects were notable during the second week. Paired comparisons between the first and second weeks revealed significant advancements in Into-Bed Time (−7.2 min), Sleep Onset Time (−9.0 min), Final Wake-up Time (−7.8 min), and Out-of-Bed Time (−7.8 min), compared to the first week. No significant changes between the first and second weeks were observed in the Non-Filter session (Fig 3). Additionally, the Filter session showed significant improvements in disruptive behavior towards siblings and friends, daytime irritability, and mood upon waking compared to the Non-Filter session. Although the sleep phase shifts were modest, they correlated with better daytime behavior. The more pronounced effects during the second week suggest that extending the study may yield larger benefits.

We initially anticipated a significant melatonin suppression in the Non-Filter session, as previous studies have demonstrated that the use of self-luminous devices starting 2 hours before normal bedtimes in adolescents (age 15–17 years) significantly reduces (~38%) salivary melatonin secretion, while wearing orange tinted glasses (SAF-T-CURE Õ Orange UV Filter Glasses) which filter all optical radiation below approximately 525 nm, reverses the melatonin suppression [36].

To our surprise, wearing partial blue light-blocking glasses advanced the sleep phase and improved daytime behavior in schoolchildren, but these effects were not mediated by changes in melatonin secretion. We used a melatonin ELISA kit (SALIMETRICS ASSAY) optimized for salivary melatonin measurements. The melatonin levels exceeded the kit’s detection limit (1.37 pg/ml), and a time-dependent increase in melatonin levels was observed during both the 1st and 2nd weeks in the Non-Filter sessions. This time-dependent increase in melatonin in the Non-Filter sessions was comparable to the changes reported previously with the non-filter control sessions for 100% blue light-blocking glass sessions [36] or in papers of the dim light melatonin onset measures, where melatonin levels were 3–4 times higher at bedtime compared to 2–3 hours before [37,38].

The suppression of melatonin by light exposure before bedtime has been reported by several research groups [30,39] and it has been suggested that younger subjects may be more sensitive to light-induced melatonin suppression [40]. Considering our negative results, it’s possible that previous studies used stronger light exposures than the light exposure in everyday life in the subjects of our study. It is also possible that the melatonin-reversing effects with blue light-blocking glasses were masked because we used partial blue light-blocking glasses, and/or melatonin supersession was only modest with Non-Filter glasses. In any case, under the daily living conditions of Japanese elementary schoolchildren, we did not observe a significant reduction in salivary melatonin levels (compared to the previous studies [30,37,38]), nor did we detect any effects of 40% partial blue light-blocking glasses on salivary melatonin secretion, even though the parents reported that the subjects used tablets for more than 1 hour at home daily. Nevertheless, we observed a significant advancement of the sleep phase and an improvement in daytime behavior, particularly during the 2nd week. In contrast, a tendency towards worsening was observed during the 2nd week of the Non-Filter sessions (Fig 3, 4). Therefore, it is likely that the advancement of the sleep phase and the improvement in daytime behavior were functionally related to the short-term use of partial blue light-blocking but were not primarily mediated by significant changes in melatonin secretion.

**Fig 5:**
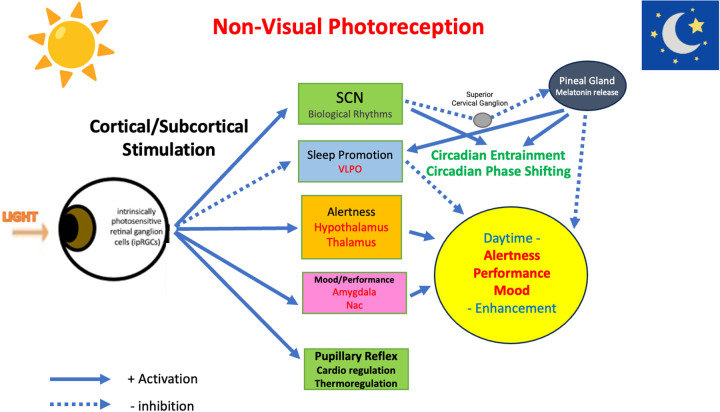
Schematic Representation of Functions of ip-RGC Non-Visual Photoreceptors.

The discovery of melanopsin (i.e., ipRGC) and its role in non-visual light perception has significantly advanced our understanding of how light affects various physiological processes beyond simple vision [17–19]. This knowledge has important implications for areas such as sleep regulation, mood disorders, and the impact of artificial light exposure on health. While one of the major mechanisms involved is thought to be mediated by melatonin secretion, there are other direct effects of blue light on the brain and body. Light signal inputs are relayed to the suprachiasmatic nucleus (SCN), which then mediates the effect on the pineal gland through the superior cervical ganglions, ultimately controlling melatonin synthesis and secretion [17–19] (see, Fig 5). Therefore, it is advisable to minimize blue light exposure at night to reduce melatonin suppression, thus avoiding circadian phase delays and sleep impairments. However, it’s important to note that daytime exposure to sunlight, including the blue light spectrum, is essential for overall human health and well-being [41]. In addition to the melatonin-mediated mechanisms, blue light itself, during daytime, has direct effects on various brain regions, including the hypothalamus, leading to circadian phase shifting and entrainment through the stimulation of clock gene expressions [19] (Fig 5). It also influences cortical activity, subjective experiences, EEG patterns, and neurobehavioral performance [42,43]. Furthermore, blue light plays a role in light-induced papillary reflex, cardiovascular regulation, and thermoregulation, all of which are mediated by ipRGC [44] (Fig 5). While melatonin-mediated effects certainly play a role in these physiological responses and the regulation of sleep and circadian rhythms, it’s possible that melatonin non-mediated effects of blue light on the brain and body may be equally important in understanding how light impacts our overall health and well-being.

Several limitations of the studies were noted. A major limitation is the inability to adjust for multiple comparisons, primarily due to the small sample size in the five-week survey of elementary school children, which resulted in low statistical power. In sleep and behavior assessments, it is common to analyze many independent variables simultaneously, and some findings may be significant by chance. To address this, we aimed for consistency in key evaluation parameters by conducting relevant assessments concurrently or by rephrasing questions. For example, advances in bed time, sleep onset time, final wake-up time, and out-of-bed time all indicate a sleep phase shift. Similarly, consistent responses regarding disruptive behaviors, daytime irritability, and poor mood upon awakening led us to believe that the results were unlikely to be due to chance.

Regarding the experimental design, we could not include dim light control sessions, as the study aimed to assess real-life effects in home settings where parents supervise procedures. It was also impractical to control and measure light exposure, as the subjects lived in, stayed in and move to various rooms in the house. Similarly, we could not control and record the light exposure history before and during the study. These limitations limit the interpretations of the melatonin results and we could not evaluate exactly and conclude if daily living evening light exposure at home reduces melatonin secretion. Secondly, the study’s blue light blocking manipulation and monitoring were conducted for only 2 weeks, with the positive effects becoming prominent in the 2nd week. It is therefore advisable to consider longer monitoring periods, as extended manipulation may reveal more significant effects, potentially including impacts on sleep duration and quality. Thirdly, the study was conducted exclusively on male children and only during early summer, limiting the generalizability of the findings to broader populations or different seasonal contexts. Finally, although our results suggest the involvement of non-melatonin pathways in sleep phase advancement and improvements in daytime behavior, the underlying mechanisms of ipRGC-mediated sleep and mood regulation remain largely unknown [19], (see also, Fig 5). More extended studies, incorporating detailed neurobehavioral monitoring of blue light blocking under daily living conditions, are warranted to gain a deeper understanding of these mechanisms.

In summary, our preliminary and exploratory study found that partial blue light-blocking glasses advanced sleep timing and reduced daytime irritability without altering salivary melatonin levels in Japanese schoolchildren. Future efforts should focus on verifying the reproducibility of these findings and conducting additional investigations that incorporate enhanced management protocols, extended monitoring periods, and detailed elucidation of the underlying mechanisms.

While it is important to emphasize that the gold standard of sleep management is teaching children to develop a proper sleep-wake cycle from infancy, our findings also suggest potential applications of blue light filters beyond children, including travelers, athletes, and shift workers experiencing circadian disruptions.

## Supporting information

S1 Fig Transmittance spectrum: comparison of blue light cut and normal lenses. (TIFF)

S2 Fig Sleep assessment report. (TIFF)

S1 File Supplement questionnaire Q1. (TIFF)

S2 File Supplement questionnaire Q2. (TIFF)

S3 File Inclusivity-in-global-research-questionnaire clean. (DOCX)

S4 File IRB research plan (COMMSMED-23-0788-T) plos one. (DOCX)
