# Supplementary figures and images for: Partial blue light blocking glasses at night advanced sleep phase and reduced daytime irritability, disruptive behavior and improved morning mood, but did not alter salivary melatonin secretion in Japanese male schoolchildren
Source: PLoS One. 2025 Oct 30;20(10):e0332877. doi: 10.1371/journal.pone.0332877 (PMC12574898; doi:10.1371/journal.pone.0332877)

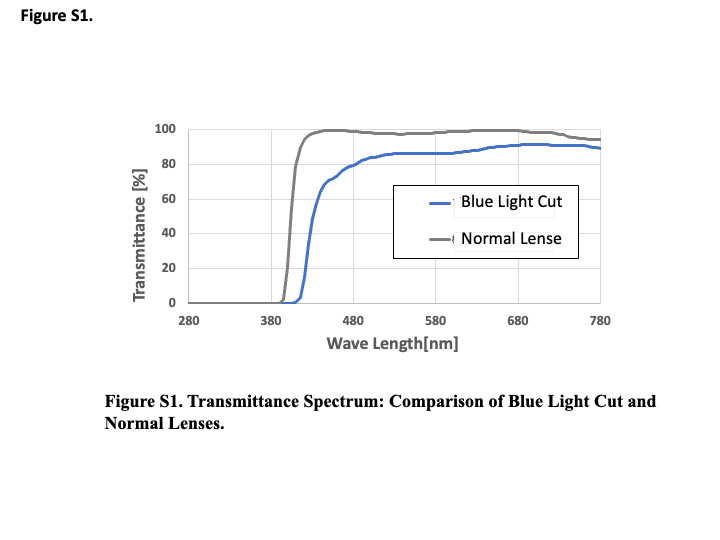

Supplement: S1 Fig — (TIFF) [file pone.0332877.s001.tiff]

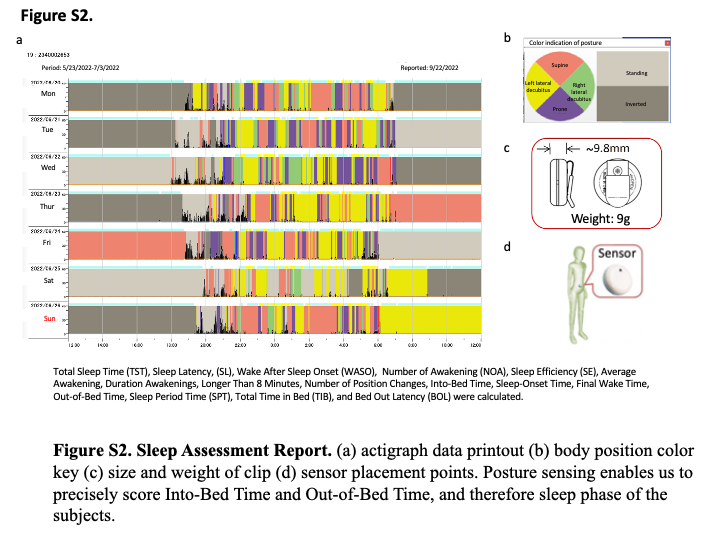

Supplement: S2 Fig — (TIFF) [file pone.0332877.s002.tiff]

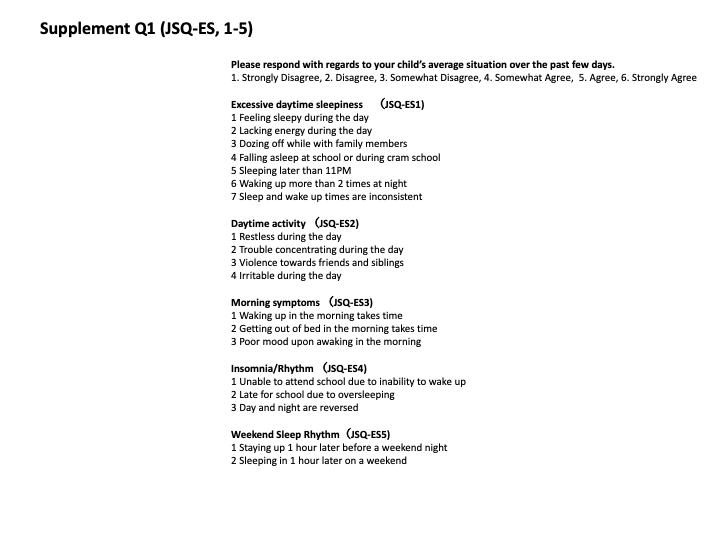

Supplement: S1 File — (TIFF) [file pone.0332877.s003.tiff]

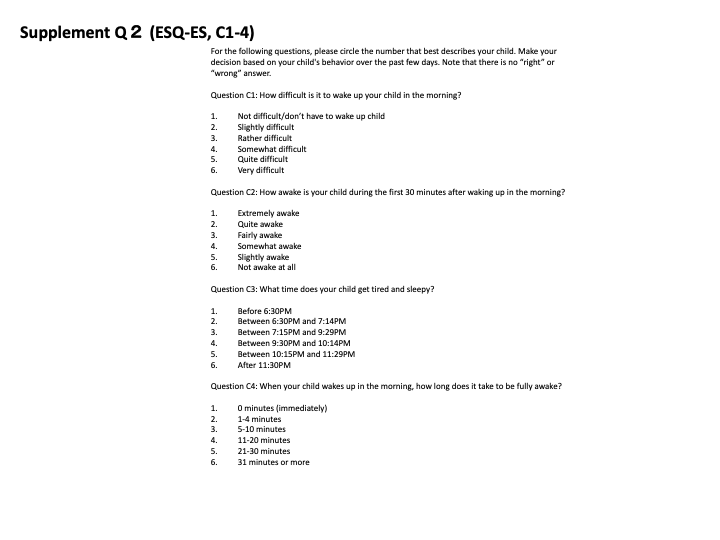

Supplement: S2 File — (TIFF) [file pone.0332877.s004.tiff]
