# Supplementary material for: Partial blue light blocking glasses at night advanced sleep phase and reduced daytime irritability, disruptive behavior and improved morning mood, but did not alter salivary melatonin secretion in Japanese male schoolchildren
Source: PLoS One. 2025 Oct 30;20(10):e0332877. doi: 10.1371/journal.pone.0332877 (PMC12574898; doi:10.1371/journal.pone.0332877)
Supplement: S4 File — (DOCX) [file pone.0332877.s006.docx]

**Notice is hereby given that the following research protocol was infringed and approved by the Ota Sleep Sciences IRB Council in March 2022 (#20028, 02/28/2022)**

**Ota Sleep Sciences IRB**

**Original Research Protocol**

**Research Plan**

**Prospective Study on the Effect of Wearing Blue Light Cut Glasses Before Bedtime on Melatonin Concentration in School Children**

**A prospective, randomized, crossover exploratory study of the effects of wearing blue light cut glasses before bedtime on melatonin levels in schoolchildren**

**Principal Investigator**

Professor, Department of Psychiatry, Stanford University School of Medicine

Director, Sleep and Biological Rhythms Laboratory, Stanford University

Director, Sleep and Biological Rhythm Laboratory, Stanford University

**Research Office**

Sleep and Biological Rhythms Laboratory, Stanford University

Version 1.0 (Creation date: February 10, 2022)

Version 1.1 (Revision date: March 15, 2022)

Version 1.2 (Revision date: April 14, 2022)

**Table of Contents**

Definitions of Abbreviations and Terms 1

1.1 Outline 1 2.

Title of Research 1 3.

Research Organization 1 4.

4. funding and equipment providers 1 5. background of the research 1

5. background of the research 1 6.

6. objectives 1 7. research design 1

7. study design 1 8.

Study Subjects and Target Number of Patients 1

9. study period 1

10. research methods 1

10.1. research procedures 1 10.1.1.

10.1.1 Schema 1

10.1.2. study schedule 1 10.1.3.

10.1.3. Study Description, Obtaining Consent, and Assent 1 10.1.4.

10.1.4. eligibility check 1

10.1.5. enrollment and allocation 1

10.1.6. investigation methods and items 1

10.2. study equipment 1 11.

Selection of Study Subjects 1 11.1.

11.1. Selection and Exclusion Criteria 1

11.2. Discontinuation Criteria 1 12.

12. evaluation items 1 12.1.

Primary Endpoints 1 12.2.

Secondary Endpoints 1

Safety evaluation 1 14.

Statistics 1 14.1.

Primary endpoint 1

Secondary endpoints 1 14.3.

Basis for setting target number of patients 1 14.4.

Significance Levels 1

15. direct access to source documents 1 16. ethical issues 1

Ethical issues 1 16.1.

Compliance with Ethical Principles 1 16.2.

Ethical Review Committee 1 16.3.

Predicted Benefits and Disadvantages to Study Subjects 1 16.4.

16.4. actions to be taken in the event of adverse health effects from the research 1 17. monitoring and auditing 1

Monitoring and auditing 1 18.

Compliance with and revision of the research protocol 1 18.1.

Discontinuation of the research 1 18.2.

18.2. revision of research protocol 1 19.

Handling of Data and Samples and Record Keeping 1 19.1.

Protection of Personal Information 1 19.2.

Data Collection 1 20.

20. publication of information 1 20.1.

Registration in Databases 1 20.2.

20.2. actions to be taken at the end of the study 1 20.3.

Arrangements for publication of results of clinical research 1 21.

Items Necessary for Appropriate Conduct of Clinical Research 1 21.1.

Conflict of Interest Status 1 21.2.

21.2. providers of funds and equipment 1

21.3. intellectual property 1 22.

22. response to consultations with research subjects and other relevant parties 1

23. references 1

24. attached document 1

Abbreviations and Definitions

Abbreviation English Japanese

ADHD Attention-Deficit Hyperactivity Disorder

CYL Cylinder Astigmatism

LED Light Emitting Diode Luminescent device

NRS Numerical Rating scale

Quality of Life Quality of Life

SPH Sphere Sphericity

UMIN University Hospital Medical Information Network

UMIN-CTR UMIN Clinical Trials Registry UMIN Clinical Trials Registry

**1. Outline of the Study**

To investigate the effects of wearing blue light-cut glasses before bedtime on melatonin levels, sleep, and diurnal rhythms in school children.

Principal Investigator Seiji Nishino, Professor, Department of Psychiatry, Stanford University School of Medicine, and Director, Sleep and Biological Rhythms Laboratory, Stanford University

Study Design Prospective, randomized, sham-controlled, single-blind, crossover, exploratory study

Study Apparatus Blue light cut glasses

Endpoints Primary endpoint: Evaluation of salivary melatonin concentration

Secondary endpoints:

1. assessment of circadian rhythm

2. assessment of sleepiness, mood, and motivation (modified version of the NRS scale for sleep)

3. assessment of sleep quality and quantity (Children's Sleep Questionnaire for Elementary School Children/Chronotype Assessment)

Study subjects

Target number of cases School children with a habit of exposure to blue light before bedtime, 40 cases

Eligibility criteria [Selection criteria

(1) Healthy elementary school boys aged 9-12 years at the time of obtaining consent

(ii) Those who regularly use glasses with vision correction in daily life

(iii) Those who are habitually exposed to blue light from LED displays, etc. for an average of one hour or more per day before bedtime.

(4) Those who have obtained written consent for participation in this study from themselves and their parents or guardians.

Exclusion criteria

(1) Those who use the following lenses, which cannot be made according to the specifications of the lenses to be used in this study, in their regular glasses in daily life.

(1) Those who use the following lenses, which cannot be made according to the specifications of the lenses used in this study, in their regular spectacles for daily use

CYL: -4.00 to 0D, distance vision SPH: 0 to +4.00D, CYL: 0 to +4.00D) or lenses other than single vision lenses such as progressive or prismatic lenses

(ii) Those who are aware that the power of the spectacles they regularly use in their daily lives is not appropriate and need to have their power re-measured at a hospital or ophthalmologist's office, etc.

(iii) Those whose lenses of eyeglasses they regularly use in their daily lives cannot be checked at eyeglass stores, etc. (iv) Those whose lenses are not suitable for sleep, autonomic nervous system, central nervous system, etc.

(iv) Those who are taking drugs or supplements that affect sleep, autonomic nerves, or central nervous system.

(v) Those who have a history of or are suffering from sleep disorder, psychiatric disease, immune/allergic disease, metabolic disorder, corneal disorder, retinal disorder, dry eye, eye strain, etc. (vi) Those who are taking medicines for hay fever, etc.

(vi) Those who need drug treatment for hay fever, etc.

(vii) Persons with infectious diseases stipulated in the Infectious Diseases Control Law and blood-borne diseases (HBV, HCV, HIV, syphilis) (including persons in close contact)

(viii) Persons who cannot properly wear the examination equipment under the supervision of a person with parental authority before going to bed.

(ix) Persons with irregular sleeping habits

(x) Persons who plan to change their living environment during participation in the research.

(xi) Any other person who is deemed inappropriate as a research subject by the physician.

Research period: From the date of approval by the Ethical Review Committee to September 2022.

Period of case registration: From the date of approval by the Ethics Review Committee to June 2022

Research Category Ethical Guidelines for Life Sciences and Medical Research Involving Human Subjects

Funds/test equipment

Provider Jinz Holdings, Inc. 2.

**Research subject**

A Prospective, Randomized, Crossover, Exploratory Study of the Effects on Melatonin Concentration in School Children Wearing Blue Light Cut Glasses Before Bedtime

3. research implementation system

Principal Investigator

Professor, Department of Psychiatry, Stanford University School of Medicine

Seiji Nishino, Director, Sleep and Biological Rhythms Laboratory, Stanford University

E-mail: nishino@stanford.edu

Research Institution and Principal Investigator

Ota Sleep Science Center, Ota General Hospital Memorial Institute Clinic, Aijinkai Medical Corporation

Director: Shintaro Chiba

Address: 1-50 Nisshin-cho, Kawasaki-ku, Kawasaki-shi, Kanagawa 210-0024, Japan

Phone: 044-244-0131 (main line)

E-mail: s.chiba3387@gmail.com

3. research office

Sleep and Biological Rhythms Laboratory, Stanford University

Taisuke Ono

E-mail: taisukeo@stanford.edu

Naoya Nishino

Email: n13@stanford.edu

Ethics Review Committee

Aijinkai Medical Corporation Ota General Hospital Ethics Committee

Address: 1-50 Nisshin-cho, Kawasaki-ku, Kawasaki-shi, Kanagawa 210-0024, Japan

5. specimen measurement institution and sleep-wake rhythm analysis institution

Specimen Measurement Institution

Yanaihara Research Institute Co.

Address: 2480-1, Awakura, Fujinomiya-shi, Shizuoka, Japan

Phone: 0544-22-2771

FAX: 0544-22-2770

Sleep/Wake Rhythm Analysis Institution

Sleep and Biological Rhythms Laboratory, Stanford University

Director: Seiji Nishino

6. person responsible for statistical analysis

Professor, Department of Psychiatry, Stanford University School of Medicine

Director, Sleep and Biological Rhythms Laboratory, Stanford University

Director, Sleep and Biological Rhythms Laboratory, Stanford University

7. research secretariat support organization

Satt Corporation

Address: ACN Shinjuku Bldg. 5F, 2-12-8 Shinjuku, Shinjuku-ku, Tokyo 160-0022, Japan

Phone: 03-5312-5026

Fax: 03-5312-5846

4. provider of funds and test equipment

Jinz Holdings Co.

Address: Iidabashi Grand Bloom 30F, 2-10-2 Fujimi, Chiyoda-ku, Tokyo 102-0071, Japan

5. background of the study

In recent years, the increase in the number of children with so-called "developmental disabilities" among school-aged children has become a social issue. According to the "Results of the Survey on the Status of Implementation of Instruction through Classroom Instruction for the Fiscal Year 2028" conducted by the Ministry of Education, Culture, Sports, Science and Technology, the number of children receiving instruction through classroom instruction is 134,185, more than double the 54,021 students in 2009, 10 years ago. The number of students with Attention Deficit Hyperactivity Disorder (ADHD) has increased particularly markedly, from 4,013 in FY2009 to 24,709 in FY 2019, an increase of more than five times. The rapid increase in ADHD, learning disabilities, autism, and emotional disorders over the past 10 years can be seen as a reflection of the loss of mental stability among children. This can be seen as a reflection of the fact that children's mental stability has been deteriorating, and it is necessary to take some measures for their healthy development.

On the other hand, the time spent on the Internet by the public, including children, has increased significantly in recent years due to faster Internet speeds and the spread of smartphones and tablet terminals. According to the "2049 Internet Use by Youth Survey (Preliminary Report)" conducted by the Cabinet Office, among the groups that responded to the survey, 35.5% of 2-year-olds use the Internet, 50.2% of 3-year-olds use the Internet, and more than 70% of 8-year-olds and older use the Internet. The average daily usage time was 67.4 minutes for 2-year-olds, 88.8 minutes for 3-year-olds, and 97.4 minutes for 8-year-olds, indicating that the main purposes are watching videos and playing games. In the results of the 2027 survey, the average duration of Internet use increased from 56.9 minutes to 97.4 minutes for 8-year-olds, for example2).

This survey on Internet use by youth began in FY2009, and in the first year of the survey, the average daily Internet use by elementary school students using computers was 24.8 minutes, and 25.5 minutes by junior high school students, while the percentage of elementary school students owning cell phones was only 21 3). In the last decade, the rate of Internet use among elementary school students has more than tripled, and the average time spent on the Internet has increased by about four times.

In light of the above findings, it is undeniable that the expansion of Internet use has shortened the sleeping hours of children, leading to a shift in their daily rhythm to a nocturnal lifestyle, which in turn has led to a decline in schoolwork and social activity, and eventually to truancy. It is also thought that biochemical factors, such as hormone disruption, may be behind this.

Viewing videos via the Internet is usually done through electronic device displays. Currently, LCD monitors are the main type of display in electronic devices, and LEDs are mainly used as the light source for backlighting. Most of the current white LEDs are used as a white light source by absorbing light from blue LEDs into a yellow phosphor to emit yellow fluorescence, which is then mixed with the original blue light. This results in the light emitted by electronic device monitors having a higher intensity of blue light, or "blue light," compared to the visible region of normal sunlight4).

Recently, it has been shown that photoreceptors that respond to the wavelength of "blue light" called ipRGCs (intrinsically photosensitive Retinal Ganglion Cells) exist in the retina of the human eye, and that nerve stimulation from these receptors causes the optic chiasmatic nucleus (OCN) in the brain to be activated by blue light. Neural stimulation from these receptors affects the production of hormones such as melatonin via the suprachiasmatic nucleus and pineal gland in the brain, and is consequently related to the human circadian rhythm4). Recent studies have also shown that there is a bidirectional relationship between ADHD and sleep disorders. In other words, sleep disorders are not only a factor in the onset of ADHD or an aggravating factor in ADHS, but are often associated with various sleep disorders in ADHD, and it has been pointed out that improvement of sleep disorders may lead to prevention of ADHD onset and improvement of symptoms5). Recently, a melatonin preparation has been approved in Japan for the improvement of sleep onset difficulties associated with childhood neurodevelopmental disorders. This is a replacement therapy that is expected to have therapeutic effects on neurodevelopmental disorders by improving sleep disturbances and diurnal rhythms through the administration of endogenous melatonin6).

Based on these findings, we believe that the disruption of children's circadian rhythms due to exposure to blue light at night from the use of smartphones and tablets may cause children to shift their lives to a nighttime pattern, preventing them from getting proper sleep, and contribute to the risk of developing mental and physical disorders in children, which have been on the increase in recent years. In this study, melatonin concentration, which is directly involved in circadian rhythm,7) will be used as an indicator of the effect on circadian rhythm, and a sham-controlled comparative study will be conducted to examine whether the use of glasses that block blue light increases melatonin concentration before and after bedtime and has a positive effect on sleep and diurnal rhythm. As a secondary evaluation, sleep and wake time will be measured with an actigraph, and sleep-related quality of life will be quantified through a questionnaire for study subjects to evaluate the effects of blue light cutoff on sleep quality, diurnal rhythm, daytime concentration, and mood.

If the effects of blue light on children's circadian rhythm and sleep quality are clarified through this study, it is expected to help in the treatment of mental and physical disorders and disorders, including ADHD, in children, which have been increasing in recent years.

6. objective

To examine whether wearing blue light-cut glasses before and after bedtime increases melatonin levels in school children who are habitually exposed to blue light before bedtime, and whether this has a positive effect on their sleep and diurnal rhythm.

7. study design

Prospective, randomized, sham-controlled, single-blind, crossover, exploratory study

8. study subjects and target number of subjects

School children with a habit of exposure to blue light before bedtime, 40 cases

9. study period

Research period: From the date of approval by the Ethical Review Committee to September 2022

Period of case enrollment: Ethics Review Committee approval date - June 2022

10. research methods

10.1 Research Procedures

10.1.1. Schema


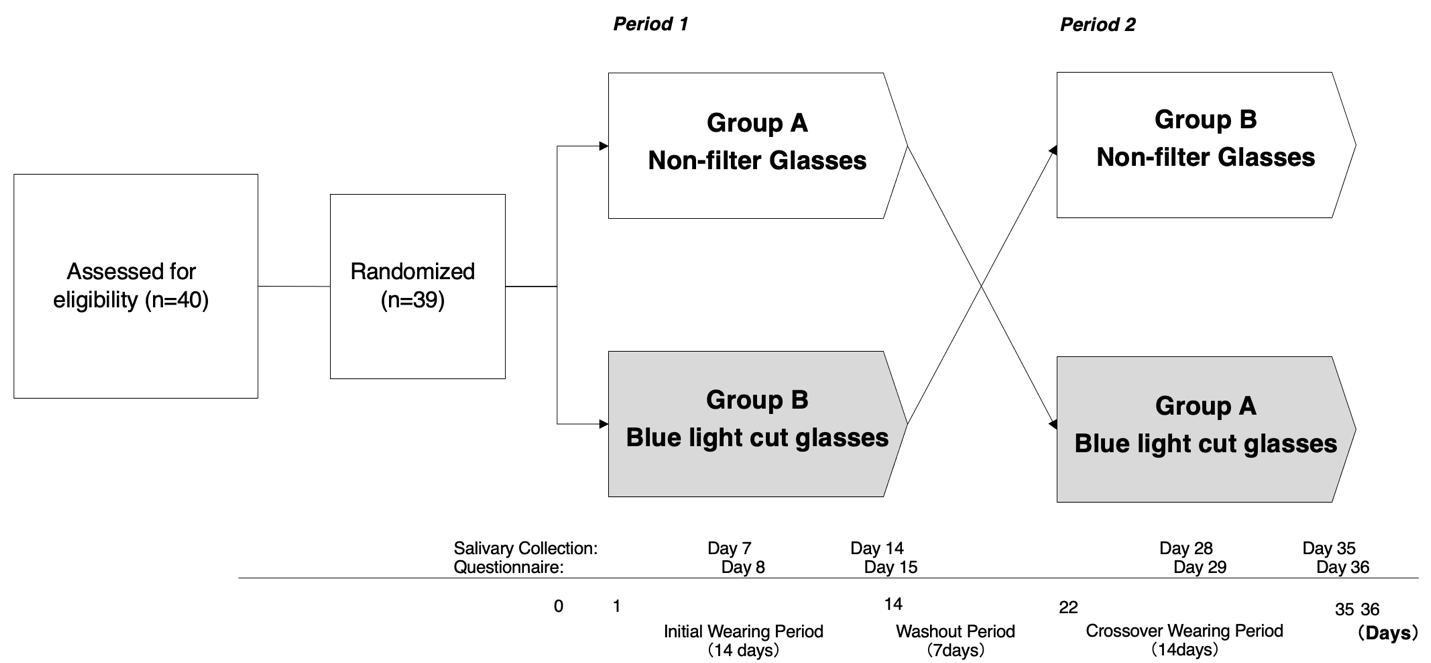


10.1.2. Study Schedule

Before the start of the study Prior wearing period Non-wearing period Switching wearing period End of the study (discontinuation)

1: Conducted voluntarily on the day before the first day of use, but no information will be collected.

2: Questionnaires will be administered by a person with parental authority to the research subjects.

3: Sleep/wake information will be collected daily at bedtime during the period of use.

10.1.3. Explanation of the study, obtaining consent, and assent

The principal investigator(s) will explain the study to the potential research subjects (school children) and their parent or guardian using the "Assent Document" (Appendix A) and the "Explanatory Document" (Appendix B), and will obtain free and voluntary consent for participation in the study from the potential research subjects (school children) themselves and their parent or guardian using the "Consent Document". Consent. In this study, the consent of only a surrogate consentor is not acceptable.

Methods of Dialogue

Prior to the confirmation of eligibility, the principal investigator (or subinvestigator) will distribute by mail the "Assent Document" for school children and the "Explanatory Document" for their parent or guardian, which have been approved by the Ethics Review Committee, to the potential research subjects themselves and their parent or guardian, and will fully explain the research orally to the participants using the web conferencing system. After explaining the research, the potential research subjects themselves will be approached to participate in the research after confirming that they have fully understood the content of the research, and the free and voluntary consent of the potential research subjects themselves and their parent or legal guardian will be obtained in writing using the "Consent Form".

If they agree to participate in the research, the potential research subject and the person with parental authority sign the consent form, enter the date of consent, and send it by mail to the physician in charge who provided the explanation. The physician in charge (including the research collaborator if the research collaborator provided supplementary explanations) will write the date of explanation and affix his/her name and seal or sign the consent form. The original consent form signed by the explainer will be kept by the principal investigator, and a copy will be mailed to the research subject. The research subject will keep a copy of the consent form sent to him/her.

If the research subject and the person with parental authority wish to withdraw consent, the research subject and the person with parental authority will fill in the date of withdrawal of consent on the "Consent Withdrawal Form" and sign it. In addition, the physician in charge and the research collaborator will fill in the date of explanation and affix their names and seals or sign the form. Two copies of the consent form and the withdrawal of consent form (if the research subject and the person with parental authority wish to withdraw consent) will be made, one copy will be mailed to the research subject and the person with parental authority, and one copy will be kept in the case file for the study.

10.1.4. Confirmation of Eligibility

The principal investigator will check the eligibility (selection and exclusion criteria) of research subjects who have given their consent to participate in this study. See "11. Selection of Research Subjects" for selection and exclusion criteria. 10.1.5.

10.1.5 Enrollment and Assignment

Study subjects will be randomly assigned to the "blue light cut glasses pre-fit group" and the "Siamese glasses pre-fit group" in a ratio of 1:1. Assignment will be made at the time of registration in the Web Registration Assignment System. Although the first pair of spectacles to be worn will be specified, the results of the individual allocation for each subject will not be communicated.

10.1.6. Survey Methods and Items

The study will be conducted in the home of the research subjects under the supervision of their custodians. Among the potential research subjects who have applied to the recruiting company, the study will be conducted on those research subjects who have given written explanation of the study to the potential research subjects, obtained written consent and assent, and meet the eligibility criteria.

During the study, the study subjects will be instructed to lead a normal life, but to disable the blue light cut mode (night mode, night mode, etc.) on the LED display electronic terminal in consideration of its impact on the study results.

The first wearing of the test spectacles will be on the 1st day and will be completed on the 14th day. The first day of wearing the test spectacles shall be Day 1 and shall be completed on Day 14, and the switching of the spectacles shall be started on Day 22 and completed on Day 35. The first day of the test (Day 1 and Day 22) should be a Friday. In addition, an optional short wearing practice session may be conducted on the day prior to Day 1.

From Day 1 to Day 14 of the prior wearing period, the blue light cut glasses prior wearing group will wear blue light cut glasses and the Siamese glasses prior wearing group will wear Siamese glasses for approximately 3 hours before bedtime every day (14 days of wearing period). Days 15 to 21 will be a non-wearing period for each group, during which the subjects will wear their regular eyeglasses. From Day 22 to Day 35 of the switching period, the subjects in the blue light-cut glasses-first group will wear Siamese glasses, and those in the Siamese glasses-first group will wear blue light-cut glasses for approximately 3 hours before bedtime every day (for 14 days). The test spectacles will be made with the same power as the spectacles that each subject wears in his/her daily life.

During the period of wearing the test spectacles, changes in sleep-wake rhythm will be recorded using an actigraph, and saliva samples will be collected by the research subjects themselves on the day before the start of wearing the spectacles, on the 7th, 14th, 28th and 35th days after the start of wearing, and questionnaires will be administered on the 8th, 15th, 29th and 36th days after the end of wearing the spectacles.

A sleep diary will be filled out daily during the period from Day 1 to Day 36.

10.1.6.1. background information on study subjects

The following items will be surveyed at the time of case enrollment to confirm eligibility

(1) Age (at the time of obtaining consent), gender, area of residence, information on the power of eyeglasses regularly used in daily life, environment of exposure to blue light, etc.

(2) Degree of sleep disturbance

The degree of sleep disturbance of each research subject will be confirmed using the Children's Sleep Questionnaire for Elementary School Children (Appendix C) by Mihoshi et al.8) . The Elementary School Children's Sleep Questionnaire was designed to assess sleep status during the last month using 9 categories (I RLS; restless legs syndrome, II excessive daytime sleepiness, III OSAS; obstructive sleep apnea syndrome, IV daytime behavior, V morning symptoms, VI Sleep duration, VII Insomnia/rhythm, VIII Sleep habits, IX Sleep rhythm on holidays). (1) Not at all applicable, (2) Not applicable, (3) Somewhat applicable, (4) Somewhat applicable, (5) Applicable, (6) Very applicable Responses within 3 months prior to case registration are considered acceptable.

10.1.6.2 Sleep/wake information

Sleep-wake rhythm information will be investigated using the following actigraph during the prior and switchover periods.

Equipment used: MicroTag activity meter MTN-221 manufactured by Accors Inc.

The subject wears the ACTIGRAPH on the abdomen with a designated belt about 3 hours before bedtime, and acts as usual until bedtime (but removes the ACTIGRAPH during bathing). The ACTIGRAPH will be worn during bedtime, removed upon waking the next day, and kept at the subject's home during the day. The belt should be the one designated for the study so that there will be no difference in the wearing condition among the study subjects.

After the ACTIGRAPH is collected by the research support organization, the measurement records will be converted into electronic data and analyzed by the Sleep-Wake Rhythm Analysis Laboratory.

10.1.6.3. Salivary melatonin concentration

Saliva samples will be collected using the following saliva collection device on the day before the start of the study, the middle day of prior use (Day 7), the end day of prior use (Day 14), the middle day of switching use (Day 28), and the end day of switching use (Day 35). Samples will then be transported to a laboratory where the melatonin concentration in saliva will be investigated using the following test instruments.

Saliva testing and collection instruments:

Salimetrix, Inc.

Melatonin test kit: SALIMETRICS ASSAY

Saliva collection straws: Saliva Collection Aid

Saliva storage container: Cryovial, 2ml, SalivaBio

Freezer box: 2" Premium Cryostorage Box

Specimen collection and shipping:

(1) The research subject should collect about 0.5 ml of saliva in a container 3 hours before bedtime, 2 hours before bedtime, 1 hour before bedtime, and just before bedtime. The collected specimens should be stored in a freezer at the research subject's home until shipping.

(2) The collected saliva should be packaged in a frozen state and transported to the specimen measurement laboratory via a carrier. Temporary storage will be conducted at a designated storage location as necessary.

(3) Saliva samples are batch-measured at the laboratory.

10.1.6.4 Research subject questionnaire

The following research subject questionnaires will be administered on the day following the middle day of prior use (Day 8), the day following the end of prior use (Day 15), the day following the middle day of switching use (Day 29), and the day following the end of switching use (Day 36).

Questionnaire items:

(1) Elementary school version of the Children's Sleep Questionnaire (excerpts)/Chronotype Assessment (Appendix C)

A total of 23 questions (6 choices), including 4 questions on I excessive daytime sleepiness, II daytime behavior, III morning symptoms, IV insomnia/rhythm, V holiday sleep rhythm, and VI chronotype in the days preceding the questionnaire response.

(2) Modified NRS Scale for Sleep (Appendix D)

The NRS will be used to evaluate the state of sleep at the time of answering the questionnaire on an 11-point scale, with 5 items: "awake, not sleepy, feeling good, motivated, and having a good appetite".

Study subjects will complete a paper questionnaire after waking up, and each will be sent by mail to the research office support organization.

The collected questionnaires will be converted into electronic data at the research secretariat support organization, and analyzed at the Sleep/Wake Rhythm Analysis Organization.

10.1.6.5. Sleep diary (information on test equipment use, sleep duration and electronic terminal use)

The following information on the use of the test equipment and other devices shall be recorded in the sleep diary (Appendix E) during the preceding and switching periods. In addition, sleep time and electronic device use time information shall be recorded in the sleep diary during the prior use, non-use, and switchover periods.

(1) Information on wearing of test equipment, etc.

The wearing time of the test spectacles will be recorded. The recorded sleep diaries and subject questionnaires will be collected on days 15 and 36, and mailed to the research secretariat support organization, respectively. The collected questionnaires will be converted into electronic data at the research secretariat support organization, and analyzed by the Sleep-Wake Rhythm Analysis Laboratory. In addition, the data from the sleep diary collected on the 15th day will be used to confirm compliance with wearing the study glasses.

(2) Sleep time and electronic device use time information

The time of sleeping and waking, time of use of devices emitting blue light, status of blue light cut mode setting (including protective film status), etc. shall be recorded.

10.2. test equipment

The following products shall be used as the test spectacles. For both blue light cut spectacles and Siamese spectacles, the power of vision correction shall be equivalent to that of the spectacles regularly used by the research subjects in their daily lives.

(1) Eyeglass frames (for both blue light cut glasses and Siamese eyeglasses)

KUF-20S-113~120 Slim Airframe-Kids Basic made by JINS

(2) Eyeglass lenses

［Blue light cut glasses]

JINS SCREEN lens HEAVY made by JINS (blue light cut rate 40%@EN standard)

［Siamese eyeglasses]

Standard clear lenses manufactured by JINS

11. selection of study subjects

11.1 Selection and Exclusion Criteria

Among school children who have a habit of being exposed to blue light before bedtime, those who meet the following selection criteria and do not violate the exclusion criteria will be included in the study.

(1) Selection criteria

(1) Healthy elementary school boys aged 9-12 years at the time of obtaining consent

(ii) Those who regularly use glasses with vision correction in daily life

(iii) Those who are habitually exposed to blue light from LED displays, etc. for an average of one hour or more per day before bedtime.

(iv) Those who have obtained written consent for participation in this study from themselves and their parent or guardian.

(2) Exclusion criteria

(1) Those who use the following lenses, which cannot be made according to the specifications of the lenses used in this study, in their regular eyeglasses in daily life.

Lenses with power that falls outside the following ranges (myopia: SPH: -8.00 to 0D, CYL: -4.00 to 0D, hyperopia: SPH: 0 to +4.00D, CYL: 0 to +4.00D) or lenses other than single vision lenses such as progressive or prismatic lenses

(ii) Those who are aware that the power of the spectacles they regularly use in their daily lives is not appropriate and need to have their power re-measured at a hospital or ophthalmologist's office, etc.

(iii) Those whose eyeglass lenses used regularly in daily life cannot be checked by eyeglass stores.

(iv) Those who are taking drugs or supplements that affect sleep, autonomic nerves, or central nervous system.

(v) Those who have a history of or are suffering from sleep disorder, mental disorder, immune/allergic disease, metabolic disorder, corneal disorder, retinal disorder, dry eye, eye strain, etc. (vi) Those who are taking medication for hay fever, etc.

(vi) Those who need drug treatment for hay fever, etc.

(vii) Persons with infectious diseases stipulated in the Infectious Diseases Control Law and blood-borne diseases (HBV, HCV, HIV, syphilis) (including persons in close contact)

(viii) Persons who cannot properly wear the examination equipment under the supervision of a person with parental authority before going to bed.

(ix) Persons with irregular sleeping habits

(x) Persons who plan to change their living environment during participation in the research.

(xi) Other subjects who are deemed inappropriate as research subjects by the physician.

11.2 Criteria for Discontinuation

The investigator will discontinue the research on the research subject in the event that any of the discontinuation criteria are met.

Discontinuation Criteria

(1) When the research subject wishes to discontinue participation in the research or withdraws consent.

(ii) In case of poor compliance in wearing the study spectacles (e.g., wearing the study spectacles for a significantly short period of time before going to bed 3 hours before bedtime)

(iii) Other cases in which the physician deems it difficult for the subject to continue the research. 12.

12. evaluation items

12.1. Main endpoints

Evaluation of salivary melatonin concentration 12.2.

12.2. Secondary endpoints

Assessment of circadian rhythm

2. evaluation of sleepiness, mood, and motivation (modified version of the NRS scale for sleep)

3. evaluation of sleep quality and quantity (Children's Sleep Questionnaire for Elementary School Children/Chronotype Assessment)

Other exploratory evaluation of effects on sleep, etc. 13.

Safety evaluation

This study will not use test instruments for the purpose of treatment, etc., but will ask research subjects to submit saliva for salivary melatonin testing. In addition, medical treatment based on the results of the questionnaire and medical treatment such as ophthalmoscopy during the preparation of the test apparatus will not be performed. In addition, the study will not assess adverse events, as the tests and questionnaires are not invasive to the research subjects, although they may be restrained. An inquiry desk will be set up for emergency inquiries from research subjects and for health-related consultations.

Statistics

14.1. primary endpoint

In the main analysis, time trends of salivary melatonin concentration (4 time points at 0, 1, 2, and 3 hours, starting 3 hours before sleep onset) were tested by repeated two-way ANOVA for Siamese eyeglass wear and blue light cut eyeglass wear (eyeglass type), and the results showed that salivary melatonin concentration was significantly higher for eyeglass type, time trend and interaction ( 14.2. Secondary Assessment Items

14.2. Secondary endpoints

(1) Evaluation of circadian rhythm

For the effects of wearing Siam glasses and blue light cut glasses on sleep and diurnal rhythm, objective sleep indices will be analyzed by automatic analysis of actigraph data, followed by visual correction under blind conditions as necessary. Sleep parameters such as time to fall asleep, time to wake up, total time to fall asleep, total sleep time, latency to fall asleep, mid-awake, and sleep efficiency are averaged from Monday to Thursday of each week and compared.

(2) Evaluation of sleepiness, mood, motivation, sleep quality and quantity

For subjective evaluation using a questionnaire, the total scores for I excessive daytime sleepiness, II daytime behavior, III morning symptoms, IV insomnia/rhythm, V holiday sleep rhythm, and VI chronotype were calculated for each week, and the data for each week for the Siam glasses wearer and blue light cut glasses wearer were compared. Statistical analysis was conducted to determine whether there was a significant change in the data with and without blue light cutoff glasses when wearing Siamese glasses or blue light cutoff glasses.

For the NRS, statistical analysis will be conducted to see if there is a significant change in each item for each week when wearing Siamese eyeglasses and when wearing blue light cut eyeglasses, using the corresponding t-tests.

Through these statistical analyses, we will examine whether wearing blue light-cut glasses before bedtime reduces exposure to blue light from smartphones and other devices, prevents the suppression of melatonin release before and after bedtime, and consequently improves sleep quality and prevents delays in the diurnal rhythm.

14.3. Rationale for setting the target number of cases

Target number of cases: Total 40 cases

(20 cases in the blue light cut glasses pre-fitting group and 20 cases in the Siamese glasses pre-fitting group)

Since there were no previous studies useful for predicting statistical power values in the current study, and since it was difficult to obtain pilot data on a small number of cases due to the nature of the experiment, we were unable to calculate power values for the number of study subjects we considered necessary for the experiment. Tentatively, 40 cases will be used as the sample size for this study, but the results of the statistical analysis will be interpreted with caution to ensure that no type I or type II errors are generated by power analysis or other means.

14.4 Significance Level

The two-tailed test shall be 5%. If multiple comparisons occur, P-values should be corrected as necessary. 15.

15. direct access to source documents

Principal investigators and research institutions will provide all clinical research-related records, including source documents, for direct inspection during investigations by ethics review committees and regulatory authorities related to clinical research.

16. ethical matters

16.1. Compliance with Ethical Principles

This research will be conducted in compliance with the research protocol, the "Ethical Guidelines for Life Sciences and Medical Research Involving Human Subjects" (Ministry of Education, Culture, Sports, Science and Technology, Ministry of Health, Labor and Welfare, and Ministry of Economy, Trade and Industry Notification No. 1, 2021), other relevant laws and regulations, and ethical principles based on the Declaration of Helsinki.

16.2. Ethical Review Committee

This research will be conducted after review and approval by the Ethical Review Committee of the institution to which the principal investigator belongs.

16.3. Anticipated Benefits and Disadvantages to Research Subjects

(1) Benefits to research subjects

 Those who wish to receive the test results (salivary melatonin concentration, circadian rhythm, etc.) will be able to receive feedback.

(2) Acknowledgments to research subjects

 As rewards to research subjects, gift certificates, etc. worth up to 24,000 yen will be offered according to the implementation of the survey, including submission of eyeglass power information, salivary melatonin test, wearing of the ACTIGRAPH, recording of sleep diaries, and responses to questionnaires.

(3) Anticipated disadvantages for research subjects

 The burden of communication fees, such as when using the web conferencing system, will be incurred.

 The subjects will be required to visit an optician to check the power of the eyeglasses they regularly use in their daily lives, which will incur transportation costs and physical restraints.

 Participation in the study requires time and physical restraints for explanation of the study, wearing the equipment, filling out the sleep diary, collecting saliva samples, and answering questionnaires (as a guide, explanation of the study: 1 hour (3 hours for joint explanation), wearing the test equipment: 3 hours/time x 28 times, filling out the sleep diary (e.g., explanation of the test: 5 minutes/time x 36 times, saliva collection: 10 minutes/time x 5 times, and answering questionnaires: 5-10 minutes/time x 4 times).

The test glasses are different from the glasses used regularly in daily life, so there is a possibility that the subjects may feel uncomfortable at the beginning. 16.4.

16.4. Response to health hazards resulting from this study

In the event of any health hazard arising from the implementation of this study, the health insurance of the research subject will be used for the treatment in principle, and appropriate treatment and other necessary measures will be taken so that the subject can receive appropriate treatment.

In this research, all persons involved in this research, including the principal investigator, will be insured by clinical research insurance in preparation for compensation for health damage caused to research subjects.

17. monitoring and auditing

Monitoring and auditing will not be conducted for this research, since it will be conducted in the research subjects' homes and is a minimally invasive research, with the exception of obtaining consent and case registration. 18.

Compliance with and revision of the research protocol

18.1. discontinuation of research

The principal investigator will consider whether or not to continue the research in the following cases

(1) Significant information is obtained concerning the quality, safety, or efficacy of test equipment used in the research

(2) When it is judged that recruitment of research subjects will be difficult and that it will be extremely difficult to achieve the target number of research subjects to be enrolled

(3) When the purpose of the research is achieved before the expected number of research subjects or the research implementation period is reached

(4) When the Ethics Review Committee has given instructions to change the research plan, etc., and it is deemed difficult to accept such instructions. 18.2.

18.2 Revision of Research Protocol

When the Principal Investigator revises the research protocol, he/she must obtain the approval of the Ethics Review Committee for the revised research protocol.

After obtaining approval for the revision, the principal investigator shall promptly provide information on the details of the revision to the principal investigators and other parties involved in the research at the medical institution where the research is conducted. 19.

19. handling of data and samples and record keeping

19.1. protection of personal information

Those involved in the implementation of this research will give due consideration to the protection of the privacy and personal information of research subjects. In conducting the research, the principal investigator will establish the necessary safety control measures and systems to protect research subject data.

Information that can identify research subjects will not be used when the results of the research are made public. Research subject data obtained in research will not be used for any purpose other than that explained when consent is obtained.

19.2 Data Collection

Questionnaires and other information submitted by research subjects will be made in a form that does not identify individuals, and will be collected by mail or other means. The research administrative support organization will keep the data in a locked cabinet for the duration of the study and transfer it to the principal investigator after the study is completed. The salivary melatonin test specimens will be submitted by the research subjects to the specimen testing laboratory via a carrier.

In addition, various data obtained from research subjects in this study will be stored at the Ota Sleep Science Center, a clinic affiliated with the Memorial Research Institute of Ota General Hospital, Aijinkai Medical Corporation, after data collection is completed. This data will be used only within the scope of the purpose for which consent was obtained.

The principal investigator shall specify the handling of missing, rejected, and abnormal data in the handling criteria to be prepared prior to data fixation.

20. publication of information

20.1 Registration in Databases

Prior to conducting a study, the Principal Investigator will register and publish an overview of the study in the UMIN Clinical Trials Registration System (UMIN-CTR) operated and managed by the University Hospital Medical Information Network (UMIN). 20.2.

20.2 Actions to be taken upon completion of the research

After the research is completed, the principal investigator of the research institution will promptly submit a report on the completion of the research to the head of the implementing medical institution.

When the head of the implementing medical institution receives the above report from the principal investigator of the research institution, he/she will submit the research termination report to the Ethics Review Committee that conducted the review of this research. 20.3.

20.3. Arrangements for publication of results of clinical research

The results obtained in this research will be published in scientific journals, etc., when they are deemed to have scientific value.

21. matters necessary for the proper conduct of clinical research

21.1. status of conflicts of interest

Decisions regarding the planning, design, conduct, analysis, and publication of this research shall be made by the Principal Investigators of this research. The Principal Investigators shall manage conflicts of interest appropriately in accordance with the conflict of interest management policies of their respective academic societies and facilities, and disclose them appropriately upon request of the academic societies and medical journals in which they plan to publish their research results.

21.2. funding and test equipment providers

This research will be funded by GINZ HOLDINGS Co. Ltd. will pay the necessary expenses to Satt Corporation, the research secretariat support organization, in accordance with the contract concluded between the two companies.

The funding and equipment provider, Zins Holdings, Inc. will not be involved in the planning, conduct, analysis, or publication of this study.

21.3. Intellectual Property

The results, data, and intellectual property rights obtained from this study will belong to the Principal Investigator. Specific handling and allocation will be determined through discussion. Whether the principal investigator's intellectual property belongs to the individual or to the research institution will be determined by the agreement of the research institution to which the principal investigator belongs. 22.

Response to consultations, etc., from research subjects, etc., and other concerned parties

Ota Sleep Science Center, Ota General Hospital Memorial Research Institute Clinic, Aijinkai Medical Corporation

Director: Shintaro Chiba

Address: 1-50 Nisshin-cho, Kawasaki-ku, Kawasaki-shi, Kanagawa 210-0024

Phone: 044-244-0131 (main line)

E-mail: s.chiba3387@gmail.com

23. References

1) "Results of the Survey on the Status of Implementation of Instruction through Classroom Instruction in the 2049 School Year (Appendix 2)," Ministry of Education, Culture, Sports, Science and Technology, 2020.

2) "2049 Survey on the Actual Conditions of Internet Use by Youth (Preliminary Report)," Cabinet Office, 2020

3) "Report on the 2009 Survey on Internet Use by Youth, Part II: Results of the Survey," Cabinet Office, 2010

4) "Effects of blue light on the circadian system and eye physiology" Gianulca T. et al. Molecular Vision 2016; 22:61-72

5) "Association of sleep disturbance with ADHD: implications for treatment" Alan Hvolby, ADHD Atten Def Hyp Disord (2015) 7: 1-18, DOI 10.1007/s12402-014-0151-0

6) Melatobel Granules for Children 0.2% Attachment (Revised June 2021)

7) "Therapeutics for Circadian Rhythm Sleep Disorders." Dodson ER, Zee PC. Sleep Med Clin. 2010;5(4):701-715 Epub 2011/01/ 19. doi: 10.1016/j.jsmc.2010.08.001.

8) Mihoshi T, Mori I, Hoshino K, Kato K, Shimizu S, Shimono K, Tachibana M, Ohno Y, Taniike M. Development of the Japanese version of the Sleep Questionnaire for Elementary School Children. Child Health Research:72(6); 789-798,2013

24. appendix

A) Assent document

B) Explanation document, consent form, and consent withdrawal form

C) Child Sleep Questionnaire for Elementary School Children, Child Sleep Questionnaire for Elementary School Children (excerpts)/Chronotype Assessment

D) Modified version of the NRS scale for sleep

E) Sleep diary
